# USP17 is required for trafficking and oncogenic signaling of mutant EGFR in NSCLC cells

**DOI:** 10.1186/s12964-018-0291-5

**Published:** 2018-11-08

**Authors:** Aidan P. McCann, Peter Smyth, Francesco Cogo, William J. McDaid, Lai Jiang, Jia Lin, Emma Evergren, Roberta E. Burden, Sandra Van Schaeybroeck, Christopher J. Scott, James F. Burrows

**Affiliations:** 1 School of Pharmacy, Queen’s University Belfast, 97 Lisburn Road, Belfast, BT9 7BL UK; 2 Centre for Cancer Research and Cell Biology, School of Medicine, Dentistry and Biomedical Sciences, Queen’s University Belfast, 97 Lisburn Road, Belfast, BT9 7BL UK

**Keywords:** Deubiquitinating, Endocytosis, Epidermal growth factor receptor, USP17, NSCLC

## Abstract

**Background:**

The deubiquitinase USP17 is overexpressed in NSCLC and has been shown to be required for the growth and motility of *EGFR wild-type* (*WT*) NSCLC cells. USP17 is also required for clathrin-mediated endocytosis of EGFR. Here, we examine the impact of USP17 depletion on the growth, as well as EGFR endocytosis and signaling, of *EGFR mutant (MT)* NSCLC cells. In particular, we examine NSCLC cells harboring an EGFR activating exon 19 deletion (HCC827), or both the L858R activating mutation and the T790M resistance gatekeeper mutation (H1975) which renders them resistant to EGFR tyrosine kinase inhibitors (TKIs).

**Methods:**

MTT, trypan blue and clonogenic assays, confocal microscopy, Western blotting and cell cycle analysis were performed.

**Results:**

USP17 depletion blocks the growth of *EGFRMT* NSCLC cells carrying either the *EGFR* exon 19 deletion, or L858R/T790M double mutation. In contrast to *EGFRWT* cells, USP17 depletion also triggers apoptosis of *EGFRMT* NSCLC cells. USP17 is required for clathrin-mediated endocytosis in these *EGFRMT* NSCLC cells, but it is not required for the internalization of the mutated EGFR receptors. Instead, USP17 depletion alters the localization of these receptors within the cell, and although it does not decrease basal EGFR activation, it potently reduces activation of Src, a key kinase in mutant EGFR-dependent tumorigenicity. Finally, we demonstrate that USP17 depletion can trigger apoptosis in *EGFRWT* NSCLC cells, when combined with the EGFR tyrosine kinase inhibitor (TKI) gefitinib.

**Conclusions:**

Our data reveals that USP17 facilitates trafficking and oncogenic signaling of mutant EGFR and indicates targeting USP17 could represent a viable therapeutic strategy in NSCLC tumours carrying either an EGFR activating mutation, or a resistance gatekeeper mutation.

## Background

The addition of ubiquitin to substrate proteins, either as a monomer, or part of a poly-ubiquitin chain, is now widely recognized as one of the most influential post-translational modifications within cells. Ubiquitination can have diverse effects on substrate proteins, including targeting them for proteasomal or lysosomal degradation, as well as influencing their protein-protein interactions, activation, or location within the cell [1]. This process can also be reversed by deubiquitinases (deubiquitinating enzymes) which remove the ubiquitin monomer, or ubiquitin chains. Six families of deubiquitinases consisting of at least 97 members have now been identified [2].

The DUB/ubiquitin specific protease 17 (USP17) family of deubiquitinases were originally identified in mice (DUB-1, DUB-1A, DUB-2) [3, 4]. The expression of the human homologue, USP17/DUB-3/Dub3 (subsequently referred to as USP17), is induced in response to cytokines, chemokines and epidermal growth factor (EGF) stimulation [5–8]. We have also shown that USP17 expression is required for proper G1 to S cell cycle progression [9] and chemokine driven (IL-8, SDF1) cell motility [7]. In addition, our group and others have shown that USP17 is overexpressed in a range of tumors when compared to normal tissue (Non-small cell lung cancer (NSCLC), ovarian, breast, colon, esophagus, cervical, osteosarcoma) [9–14]. USP17 expression levels are also associated with poor prognosis and metastases in NSCLC, osteosarcoma and ovarian tumours [12–14]. Moreover, USP17 has been proposed as a potential therapeutic target as its depletion can inhibit the growth and migration of multiple cancer cell types, as well as in vivo tumour models [7, 9–16].

Epidermal growth factor receptor (EGFR) is over-expressed in many NSCLCs (~ 60%) and tyrosine kinase activating mutations of EGFR are present in a significant proportion of patients (10% in Caucasian populations and 30–40% in Asian populations) [17], and of these mutations ~ 90% are either a deletion of exon 19 (in-frame), or a point mutation in exon 21 (L858R) [18, 19]. This led to the development of a range of EGFR tyrosine kinase inhibitors (TKIs) (e.g. erlotinib, gefitinib), and other agents such as cetuximab, aimed at inhibiting EGFR [20]. EGFR TKIs are effective against tumors exhibiting EGFR activating mutations, with a response rate of 60–80% [20]. However, these patients rapidly develop resistance, rendering these drugs ineffective within 1–2 years [20]. There are a wide range of resistance mechanisms to EGFR inhibition identified, but in NSCLC it is mainly due to the acquisition of the EGFR T790M gatekeeper mutation (~ 60%) in the ATP binding pocket of the EGFR kinase domain, with the second most common resistance mechanism being the amplification of the hepatocyte growth factor receptor (MET) gene (10–20%) [17]. Second generation EGFR TKIs, such as afatinib, initially showed promising results against cells bearing the EGFR T790M mutation, but ultimately proved no more effective than first generation drugs [21]. However, third-generation EGFR TKIs, such as osimertinib, now appear to be able to overcome the EGFR T790M mutation, although combination trials trying to overcome other resistance mechanisms have so far failed [21]. Therefore, the identification of alternate targets which can help a larger proportion of NSCLC patients, complement the EGFR TKIs, or combat EGFR TKI resistance, are still a major priority.

Previously, we demonstrated that USP17 expression is required for clathrin-mediated endocytosis (CME) of EGFR [8]. As mutant EGFRs are constitutively internalized [22, 23] and appear to preferentially undergo CME [22], we hypothesized that blocking CME of mutant EGFRs by depleting USP17 would impede their internalisation and oncogenic signaling. Other studies have shown that blocking EGFR endocytosis enhances the efficacy of the EGFR TKI gefitinib in *EGFRWT* NSCLC cells [24]. Suppression of EGFR endocytosis, when combined with gefitinib, significantly inhibited in vitro and in vivo growth of *EGFRWT* NSCLC cells, and prompted a large proportion of *EGFRWT* NSCLC cells to undergo apoptosis [24]. Therefore, we also hypothesized that blocking EGFR CME in NSCLC cells by depleting USP17 could enhance the efficacy of gefitinib in *EGFRWT* NSCLC cells.

In this study, we demonstrate that USP17 depletion blocks the growth of NSCLC cells which express activated and EGFR TKI resistant EGFR mutants. In addition, although USP17 depletion does block CME in these cells, it does not block internalisation of the EGFR mutants, even though it does alter their downstream signaling. We also demonstrate that USP17 depletion preferentially triggers apoptosis in NSCLC cells that bear EGFR activating mutations. Finally, we show that USP17 depletion can enhance the efficacy of EGFR TKIs toward *EGFRWT* NSCLC cells and trigger apoptosis of these cells. This data indicates USP17 represents a potentially exciting therapeutic target in *EGFRMT* NSCLC tumors, even those that have developed EGFR TKI resistance. In addition, in combination with EGFR TKIs, targeting USP17 can also potentially be used to treat *EGFRWT* NSCLC tumors.

## Methods

### Materials

Gefitinib (ZD1839) was purchased from SelleckChem (Suffolk, UK). Biotinylated transferrin was purchased from Sigma.

### Plasmids

The pSUPER-USP17shRNA (USP17 shRNA1; target sequence 5’-GCAGGAAGATGCCCATGAA-3′), pRS-USP17shRNA (USP17 shRNA2; target sequence 5’-GATGATTTGGCTCCTGTGGCAAGACAGCT-3′) and pRS-scrambled shRNA were previously described [7, 8].

### Cell culture and DNA transfections

A549 cells (American Type Culture Collection (ATCC), Manassas, USA) were grown in DMEM supplemented with 10% FCS, 1% penicillin (10,000 U/ml) /streptomycin (10,000 μg/ml), and 1% L-glutamine (200 mM) (Life Technologies-Gibco, Paisley, UK). H1975 and HCC827 cells (American Type Culture Collection (ATCC), Manassas, USA) were grown in RPMI-1640 supplemented with 10% FCS, 1% penicillin (10,000 U/ml) /streptomycin (10,000 μg/ml), and 1% L-glutamine (200 mM) (Life Technologies-Gibco, Paisley, UK). Cells lines were grown at 37 °C in a 5% CO_2_ humidified incubator.

Cells were transfected with Xtreme-GENE HP ™ transfection reagent (Roche Diagnostics, Indianapolis, USA) according to manufacturer’s instructions. Cells were seeded between 0.5 × 10^6^ and 1.0 × 10^6^ cells for cell cycle analysis or protein experiments or 0.7–2.5 × 10^4^ on 4-well glass culture slides (BD Falcon, Bedford, USA) for microscopy experiments. The cells were transfected with 2 μg of plasmid DNA for protein experiments and biological assays or 0.25 μg of plasmid DNA for confocal microscopy experiments. For those experiments with EGF stimulation, cells were rested for 3 h in DMEM medium without serum. Cells were then stimulated with 0.32 nM recombinant human EGF (Invitrogen-Gibco, Maryland, USA) for the indicated times in the figures, corresponding to the low (2 ng/mL) EGF concentrations previously used [8, 25].

### Confocal microscopy

Cells were seeded at 0.7–2.5 × 10^4^ cells/1.7 cm^2^ well of glass culture slides (BD Falcon, Bedford, USA). Cells were transfected as previously described. The cells were fixed in 4% parafomaldehyde (Sigma-Aldrich, Steinheim, Germany), in PBS for 20 min. The cells were then permeabilized in 0.5% Triton X-100 in PBS for 5 min, washed in PBS and blocked in blocking solution (1% BSA, 10% donkey serum [both from Sigma, St. Louis, USA] in PBS) for 1 h at RT. Transfected proteins and cell organelles were stained with appropriate antibodies or counter stains according to manufacturer’s protocol. Antibodies and co-stains were as follows: mouse anti-EGFR (GR01L, 1:1000, Merck-Calbiochem, Darmstadt, Germany), mouse anti-transferrin receptor (1:100, Invitrogen, Camarillo, USA), donkey anti-mouse Alexa Fluor 488 (1:200, Invitrogen-Molecular Probes, Eugene, USA). The slides were sealed with a coverslip and Prolong Gold antifade mounting media with DAPI (Life Technologies-Molecular Probes, Eugene, USA). Slides were viewed on a Leica SP8 Confocal Microscope. Fluorescent images were captured with a 63x lens zoomed 1-4x with a 1024 × 1024 frame and 400 Hz scanning speed. Images were analyzed using Leica LAS X software. The images presented in the same figures were captured using standardized setting and exposure times.

### FACS analysis

Cells were incubated with anti-EGFR monoclonal antibody (1:50; BD Biosciences) for 60 min at 4 °C. A mouse IgG1 (BD Biosciences) was used as isotype-matched control. Cell surface expression samples were analysed by FACS Acuri Plus (BD Biosciences). Apoptosis was evaluated using propidium iodide (PI) staining [26]. Cells were trypsinized, fixed in ethanol for 1 h at 4 °C, stained with propidium iodide (PI) solution (10 μg/mL) with RNase A (250 μg/mL), incubated at 37 °C for 30 min, and analysed by FACS Calibur (BD Biosciences) and FlowJo software.

### RNA extraction and reverse transcription-PCR

RNA was extracted using STAT-60 according to the manufacturer’s instructions (Tel-Test Inc., Friendswood, USA). Reverse transcription-PCR (RT-PCR) was performed on 1 μg of total RNA using ImProm-II Reverse Transcription System (Promega, Madison, USA) as described previously [8]. The following primers were used: USP17, 5′-CAGTGAATTCGTGGGAATGGAGGACGACTCACTCTAC-3′ (forward) and 5′-AGTCATCGATCTGGCACACAAGCATAGCCCTC-3′ (reverse). B2M 5’-GTATGCCTGCCGTGTGAAC-3′ (forward) and 5′- AAAGCAAGCAAGCAGAATTTGG-3′ (reverse).

### Cell lysis and immunoblotting

Cells were lysed in the following buffer: 25 mM TrisHCl pH 7.6, 150 mM NaCl, 1% NP-40, 1% sodium deoxycholate, 0.1% SDS, supplemented with phenylmethylsulphonyl fluoride (1 mM), aprotinin (1.7 μg/ml) and leupeptin (10 μg/ml). Lysates were left on ice for 20 mins, centrifuged at 15,000 x g for 10 min at 4 °C. Equal volumes of whole cell lysate were added to Laemlli buffer to a final concentration of 1X with 5% β-mercaptoethanol (Sigma, Germany). The samples were boiled for 5 min at 99 °C for protein denaturation. The samples were analyzed by SDS-PAGE and Western blotting on PVDF membrane (Millipore, Waterford, UK). The membranes were then blocked in appropriate blocking agent, either 5% marvel or 3% BSA, in 0.1% Tween-20/PBS for 1 h. After blocking, the membranes were probed with the indicated antibodies for 1 h at RT or overnight at 4 °C. The following primary antibodies were used: rat anti-tubulin (1:10000, Abcam, Cambridge, UK), mouse anti-ERK, mouse anti-pERK1/2 (Thr202/Tyr204), rabbit anti-Src, rabbit anti-pSrc (Tyr416), rabbit anti-AKT, rabbit anti-pAKT (Ser473), rabbit anti-pEGFR (Tyr1068), rabbit anti-cleaved PARP, rabbit anti-cleaved caspase 3, mouse anti-caspase 9, rabbit anti-BCL-2 (1:1000, Cell Signaling, Danvers, USA), mouse anti-EGFR (BD Biosciences, USA). The membrane was incubated with the appropriate secondary antibody: either goat anti-mouse HRP conjugate or goat anti-rabbit HRP conjugate (both diluted 1:10,000, BioRad, Hertfordshire, UK) or rabbit anti-rat HRP conjugate (1:40,000, Abcam, Cambridge,UK). Proteins were detected with a chemiluminescence protocol and were exposed using the ChemiDoc XRS+ imaging system (BioRad, Hercules, USA).

### MTT assay

Cell viability was determined using 3-(4,5-dimethylthiazol-2-yl)-2,5-diphenyltetrazolium bromide (Sigma) [27]. Representative results of at least 3 independent experiments are shown.

### Caspase activity assay

Caspase activity was measured by a fluorogenic substrate assay. Ac-DEVD-AMC substrate (Enzo) were used to detect the activity of caspase 3/7, according to manufacturer’s instructions.

### Clonogenic survival assay

To determine the effects of USP17 depletion on cell proliferation, cells were transfected as described. The following day, transfected cells were trypsinised and reseeded on 6-well plates. After 72 h, the growth medium was replaced with fresh medium and cells were allowed to grow for a further 11 days. Colonies were visualized by crystal violet staining. Representative results of at least 3 independent experiments are shown.

### Statistical analysis

Student’s t-tests and 2-way ANOVA were calculated using the GraphPad software (Prism5). 2-way ANOVA test was used to determine the significance of changes in localisation, cell number, cell viability, and clonogenic survival, and were described as significant having *P* values that were considered significant * < 0.05, ** < 0.01, *** < 0.001. *P* values of > 0.05 were considered non-significant (ns).

## Results

### USP17 is required for NSCLC cell proliferation and colony formation, independent of the *EGFR* mutational status

USP17 is overexpressed in NSCLC and its expression levels are associated with poor prognosis and metastases [13]. USP17 depletion has also been shown to inhibit the growth and migration of NSCLC cells, suggesting it represents a potential therapeutic target in NSCLC [11]. However, this study only examined *EGFRWT* NSCLC cells and did not assess the impact of USP17 in cells with EGFR activating mutations [11]. Therefore, as we have shown that USP17 is required for EGFR CME [8], and mutant EGFR preferentially undergoes CME promoting its recycling and its oncogenic activity [22], we wanted to examine if the impact of USP17 depletion would be more potent in *EGFRMT* NSCLC cells.

**Fig. 1:**
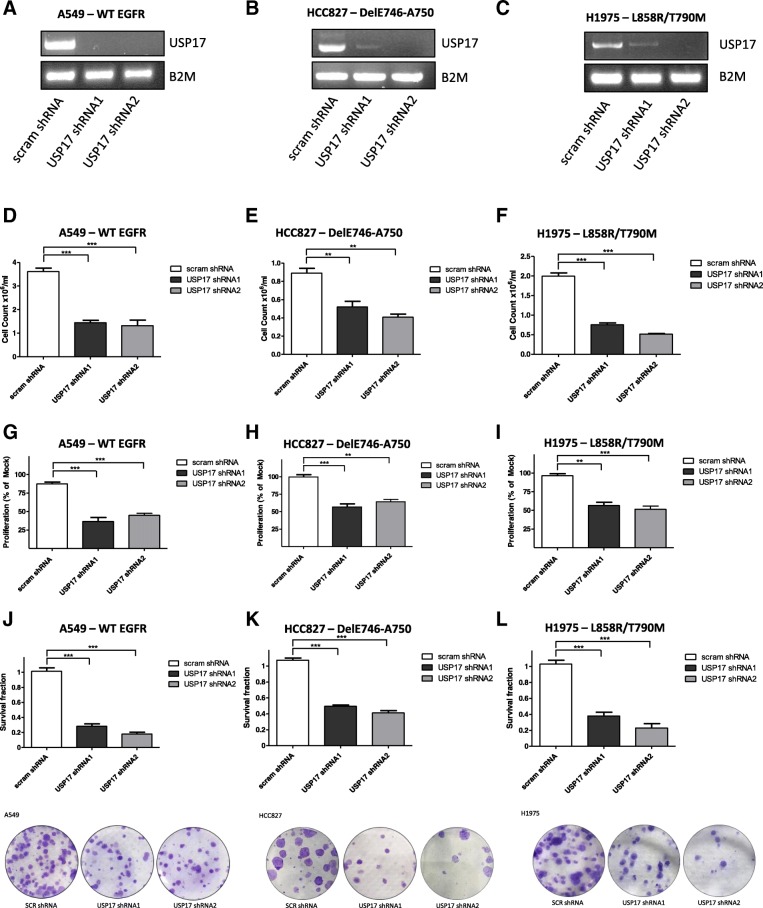
(**a**-**c**) A549, HCC827, and H1975 cells were transfected as indicated and 72 h post-transfection USP17 mRNA expression was assessed by RT-PCR. B2M mRNA expression was also assessed as a loading control. (**d**-**f**) Equivalent numbers of A549, HCC827, and H1975 cells were transfected as indicated and 72 h post-transfection, the number of viable cells was determined by trypan-blue exclusion assay. (**g**-**i**) Equivalent numbers of A549, HCC827, and H1975 cells were transfected as indicated and 72 h post-transfection, cell viability was determined by MTT assay and the results reported as % of control. (**j**-**l**) A549, HCC827, and H1975 cells were transfected as indicated and 24 h post-transfection, equivalent numbers of cells were re-plated and subsequently incubated for a further 11 days. Colony numbers were then assessed and plotted as the survival fraction in comparison to control. Representative images are included in the bottom panels. ** *p* < 0.01, *** *p* < 0.001

To assess the impact of USP17 on NSCLC proliferation, we transfected A549 (*EGFRWT*), HCC827 (DelE746-A750) and H1975 (L858R/T790M) cells with either of two validated USP17 specific shRNAs (shRNA1 and shRNA2) (Fig. 1A/B/C) [7–9] or a non-targeting shRNA. The impact on cell viability of depleting USP17 was examined by MTT and trypan-blue exclusion assay. USP17 depletion significantly reduced A549 (Fig. 1D, G), HCC827 (Fig. 1E, H) and H1975 (Fig. 1F, I) cell numbers and viability, indicating that USP17 is required for the proliferation of NSCLC cells independent of the *EGFR* mutational status.

We next examined the impact of USP17 depletion on colony formation (Fig. 1J-L). Survival fractions were calculated using the non-targeting shRNA control as 100%. It was clear that USP17 depletion significantly reduced the ability of all the NSCLC cells (A549, HCC827, H1975) (Fig. 1J-L) to form colonies. Again, this indicates USP17 depletion reduces proliferation independent of the *EGFR* mutational status.

### USP17 alters the localization of mutant EGFRs in NSCLC cells

**Fig. 2:**
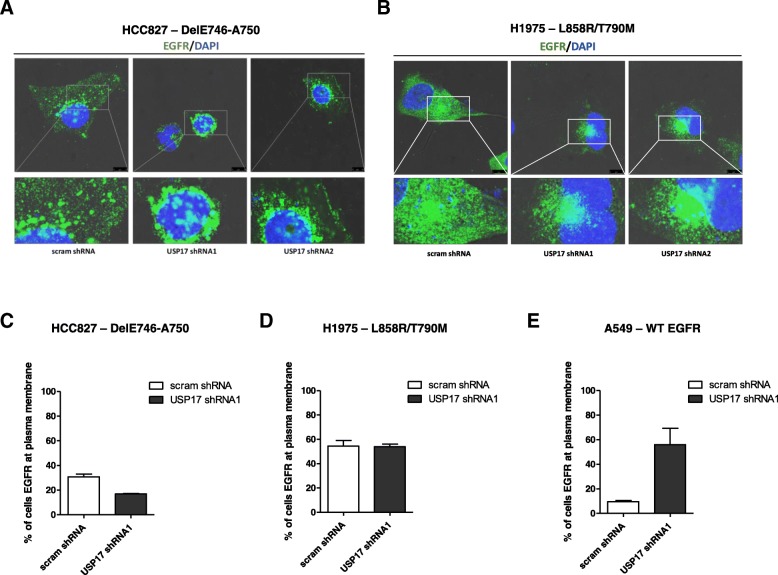
(**a**) HCC827 cells were transfected as indicated. 72 h post transfection the cells were starved in serum free medium for 3 h prior to incubation with 0.32 nM recombinant EGF. After 15 min the cells were fixed and stained using an anti-EGFR antibody (green) and EGFR localisation was assessed in brightfield and fluorescent images taken using confocal microscopy. Bottom panels are enlarged images of the indicated area in the upper panels. Scale bar = 10 μm. (**b**) H1975 cells were transfected as indicated. 72 h post transfection the cells were starved in serum free medium for 3 h prior to incubation with 0.32 nM (2 ng/ml) recombinant EGF. After 15 min the cells were fixed and stained using an anti-EGFR antibody (green) and EGFR localisation was assessed in brightfield and fluorescent images taken using confocal microscopy. Bottom panels are enlarged images of the indicated area in the upper panels. Scale bar = 10 μm. (**c**) HCC827 cells were transfected as indicated and 72 h post-transfection the cells were serum starved in serum free medium for 3 h. Subsequently the cells were incubated with 0.32 nM recombinant EGF and after 15 min, the cells were washed and stained for EGFR using an anti-EGFR FITC, for 30 min at 4 °C. After incubation cells were washed and EGFR cell membrane expression assessed by flow cytometry using receptor-specific FITC-conjugated mAbs. Expression was compared with a nonspecific isotype-matched control antibody. Percent of cells with fluorescent EGFR was plotted as a representative histogram. (**d**) H1975 cells were transfected as indicated. 72 h post-transfection the cells were serum starved in serum free medium for 3 h. Subsequently the cells were incubated with 0.32 nM recombinant EGF and after 15 min incubation, cells were washed and stained for EGFR using an anti-EGFR FITC, for 30 min at 4 °C. After incubation cells were washed and EGFR cell membrane expression assessed by flow cytometry using receptor-specific FITC-conjugated mAbs. Expression was compared with a nonspecific isotype-matched control antibody. Percent of cells with fluorescent EGFR was plotted as representative histograms. (**e**) A549 cells were transfected as indicated. 72 h post-transfection the cells were serum starved in serum free medium for 3 h. Subsequently the cells were incubated with 0.32 nM recombinant EGF and after 15 min incubation, cells were washed and stained for EGFR using an anti-EGFR FITC, for 30 min at 4 °C. After incubation cells were washed and EGFR cell membrane expression assessed by flow cytometry using receptor-specific FITC-conjugated mAbs. Expression was compared with a nonspecific isotype-matched control antibody. Percent of cells with fluorescent EGFR was plotted as representative histograms

We have previously demonstrated that USP17 is required for EGFR CME [8]. However, although it is known that mutant EGFRs constitutively internalize and preferentially undergo CME [22], it is unclear what role USP17 plays. Therefore, to investigate if USP17 is required for the endocytosis and trafficking of mutant EGFRs in NSCLC cells we transfected the HCC827 and H1975 cells with shRNAs targeting USP17 and examined the localisation of EGFR using an anti-EGFR antibody after 15 min 2 ng/ml EGF treatment (Fig. 2). EGFR subjected to low concentrations (< 2 ng/ml) of EGF are internalized via CME and this is the physiologically relevant concentration in tumours [28]. In both the HCC827 (Fig. 2A, C) and H1975 (Fig. 2B, D) cells, EGFR was observed on the plasma membrane and intracellular vesicles throughout the cell in the control cells. Unexpectedly, upon USP17 depletion, in both the HCC827 (Fig. 2A, C) and H1975 (Fig. 2B, D) cells, EGFR was still predominantly observed on intracellular vesicles, as well as the plasma membrane. However, the location of the vesicles bearing EGFR in both the HCC827 and H1975 cells appeared to shift to a more perinuclear localization upon USP17 depletion (Fig. 2A-B). This perinuclear localisation was remarkably similar to that observed in USP17 depleted cells treated with high EGF concentrations (> 2 ng/ml) which bypass the CME block by instead using clathrin-independent endocytosis routes [8]. The observation that these EGFR mutants were not trapped at the cell surface upon USP17 depletion was also confirmed by staining non-permeabilised cells for cell surface EGFR and analyzing them by flow cytometry (Fig. 2C-D). In addition, we transfected A549 (*EGFRWT*) as indicated and stained non-permeabilised cells for cell surface EGFR to confirm USP17 depletion blocked EGFR CME (Fig. 2E). As reported previously [8], USP17 depletion caused a marked re-localisation of EGFR to the PM in these cells.

**Fig. 3:**
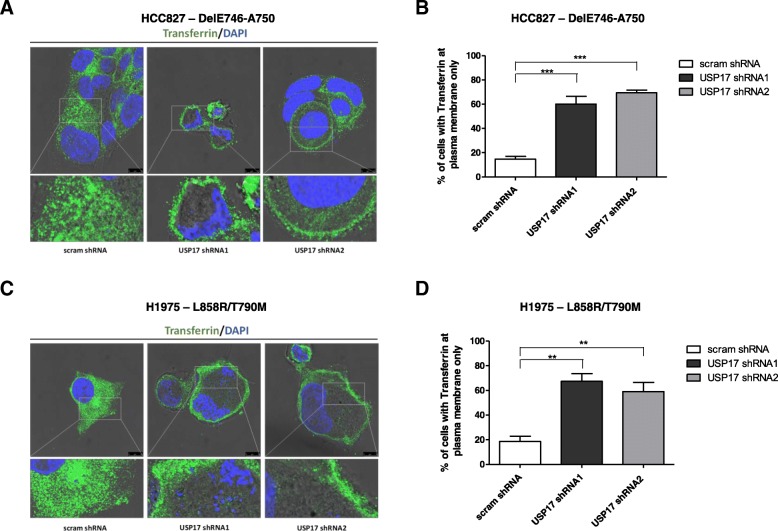
(**a**) HCC827 cells were transfected as indicated and 72 h post transfection the cells were starved in serum free media for 30 mins prior to incubation with 25 μg/mL transferrin (Sigma). After 15 min the cells were fixed and stained using an anti-transferrin antibody (green) and transferrin localisation was assessed in brightfield and fluorescent images taken using confocal microscopy. Bottom panels are enlarged images of the indicated area in the upper panels. Scale bar = 10 μm. (**b**) At least 50 cells per condition were blindly scored for three separate experiments based on the plasma membrane localisation of transferrin receptor. (**c**) H1975 cells were transfected as indicated and 72 h post transfection the cells were starved in serum free media for 30 mins prior to incubation with 25 μg/mL transferrin (Sigma). After 15 min the cells were fixed and stained using an anti-transferrin antibody (green) and transferrin localisation was assessed in brightfield and fluorescent images taken using confocal microscopy. Bottom panels are enlarged images of the indicated area in the upper panels. Scale bar = 10 μm. (**d**) At least 50 cells per condition were blindly scored for three separate experiments based on the plasma membrane localisation of transferrin receptor. ** *p* < 0.01, *** *p* < 0.001

Therefore, to confirm that USP17 depletion was actually blocking CME in the HCC827 and H1975 cells, we next examined the localisation of transferrin receptor (TfR), which is internalized almost exclusively by CME, using an anti-TfR antibody (Fig. 3). In both HCC827 (Fig. 3A-B) and H1975 (Fig. 3C-D) cells, TfR was predominantly observed on intracellular vesicles in the controls cells. However, upon USP17 depletion, TfR in both the HCC827 (Fig. 3A-B) and H1975 (Fig. 3C-D) cells was now observed predominantly at the plasma membrane. As TfR can only internalize via CME, this confirmed that USP17 depletion blocks CME in HCC827 and H1975 cells. Therefore, this indicated that USP17 depletion was blocking CME in both cell lines, but rather than being trapped at the cell surface like wild type EGFR, the mutants shift to an alternative endocytosis pathway, allowing them to traffic to endosomes where they can trigger intracellular signaling.

### USP17 is required for Src activation by mutant EGFR

The observation that USP17 depletion in the HCC827 and H1975 cells resulted in altered mutant EGFR trafficking led us to examine if this was altering the downstream signaling from these mutant receptors.

To probe the impact of USP17 depletion upon the signaling of the EGFR mutants, we knocked down the expression of USP17 in HCC827 and H1975 cells and serum starved for 3 h to minimize potential signaling crosstalk from sources other than the constitutively active EGFR mutants. Cell lysates were separated by SDS-PAGE and immunoblotted to determine the phosphorylation status of EGFR (Tyr1068), Akt (Ser473), ERK1/2 (Thr 202/Tyr204) and Src (Tyr416). Total protein levels were also assessed to determine if USP17 depletion impacted upon the stability of each protein.

**Fig. 4:**
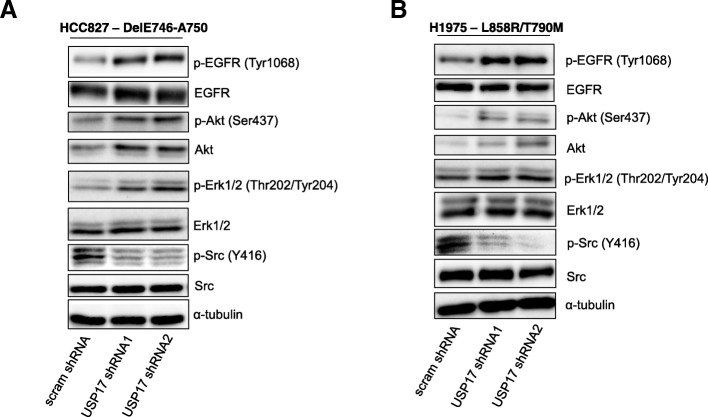
(**a**) HCC827 cells were transfected as indicated. 72 h post transfection the cells were starved in serum free medium for 3 h. Whole cell lysates were harvested and levels of phosphorylated Erk1/2, Akt, Src and EGFR were assessed by immuno-blotting using anti-pERK1/2, anti-pAkt, anti-pSrc and, anti-pEGFR (Tyr1068). Total protein levels of Erk, Akt, Src and EGFR were also assessed in addition to α-tubulin, utilising Erk1/2, Akt, Src, EGFR and anti-tubulin antibodies. (**b**) H1975 cells were transfected as indicated. 72 h post transfection the cells were starved in serum free medium for 3 h. Whole cell lysates were harvested and levels of phosphorylated Erk1/2, Akt, Src and EGFR were assessed as before. Total protein levels of Erk, Akt, Src and EGFR were also assessed in addition to α-tubulin, as before

USP17 depletion was found to enhance the activation of EGFR, Akt and ERK1/2 in NSCLC cells expressing both EGFR DelE746-A750 (Fig. 4A) and EGFR L858R/T790 M (Fig. 4B). This correlated with our previous finding that USP17 depletion prolonged ERK1/2 activation upon stimulation of wild type EGFR [8] and indicates USP17 expression is not required for EGFR activation. In addition, as USP17 depletion does not impact upon EGFR protein levels, it also indicates its depletion does not reroute mutant EGFR receptors to the lysosome for degradation. USP17 depletion had little impact upon the protein levels of EGFR and ERK1/2, but Akt protein levels increased upon USP17 depletion in both cell lines (Fig. 4A-B). In contrast, Src (Tyr416) activation was dramatically reduced upon USP17 depletion in both HCC827 and H1975 cells, whilst Src protein levels were unaffected (Fig. 4A-B).

Taken together, this data clearly demonstrated that these EGFR mutants remained active in the absence of USP17, but that their signaling profile was altered, probably as a result of the alternative trafficking.

### USP17 depletion triggers apoptosis in NSCLC cells with EGFR mutations

**Fig. 5:**
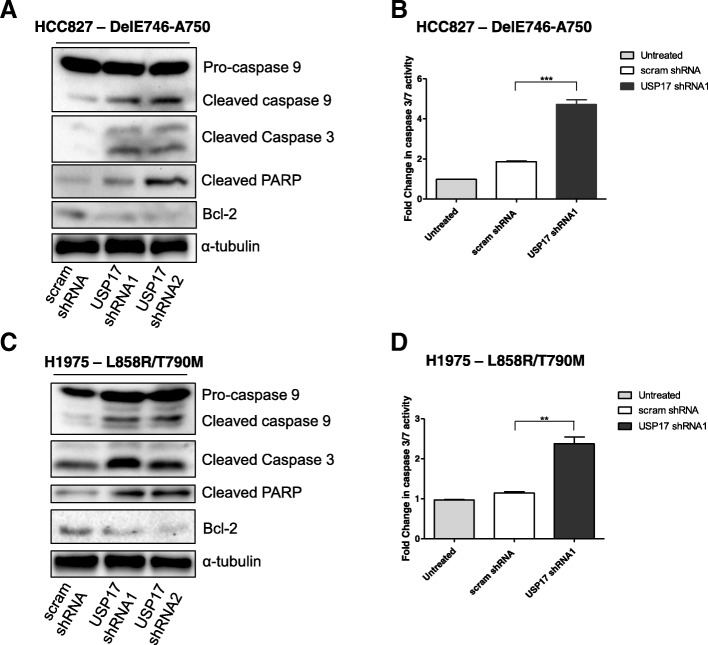
(**a**/**c**) HCC827 and H1975 cells were transfected as indicated. 48 h post transfection, whole cell lysates were harvested and levels of cleaved PARP, cleaved caspase 9, cleaved caspase 3, and BCL-2 were assessed by immuno-blotting using anti-cleaved PARP, anti-caspase 9, anti-cleaved caspase 3, anti-BCL-2, in addition to α-tubulin, as a loading control. (**b**/**d**) HCC827 and H1975 cells were transfected as indicated. Caspase-3/7 activity levels were assessed 48 h post-transfection. ** *p* < 0.01, *** *p* < 0.001

Our initial experiments demonstrated a reduction in the viability of the *EGFRMT* NSCLC cells upon USP17 depletion (Fig. 1), but the assays used didn’t specify between a cytostatic or cytotoxic effect. Therefore, as inhibition of Src in EGFR-dependent NSCLC cell lines has previously been shown to induce apoptosis [29], we hypothesized that the observed reduction in Src activation (Fig. 4) could be triggering a similar effect. We transfected HCC827 and H1975 cells as before and immunoblotted lysates taken after 48 h for markers of apoptosis, specifically those which Src has previously been shown to regulate [30–34]. Immunoblotting revealed that when USP17 is depleted in HCC827 and H1975 cells, levels of cleaved PARP, caspase 9 and caspase 3 (Fig. 5A, C), as well as caspase 3/7 activity (Fig. 5B, D), increased. In addition, the levels of the anti-apoptotic protein BCL-2 were reduced, signifying a reduction in the apoptotic threshold of these cells. (Fig. 5A, C). These results indicated that, in both the HCC827 and H1975 cells, depletion of USP17 triggered the initiation of apoptosis, something which correlates with the observed reduction in Src activation.

### USP17 depletion results in apoptosis when combined with EGFR TKIs in *EGFRWT* NSCLC cells

EGFR TKIs have been shown to be effective against tumours exhibiting EGFR activating mutations, but lack efficacy in tumours without these mutations [20]. However, blocking both clathrin-dependent and clathrin-independent endocytosis has previously been shown to enhance the efficacy of the EGFR TKI gefitinib towards NSCLC cells expressing *EGFRWT*, and prompt increased levels of apoptosis when combined with gefitinib treatment [24]. Therefore, we wanted to determine if blocking CME in *EGFRWT* NSCLC cells by depleting USP17 could also enhance the efficacy of gefitinib towards these cells.

**Fig. 6:**
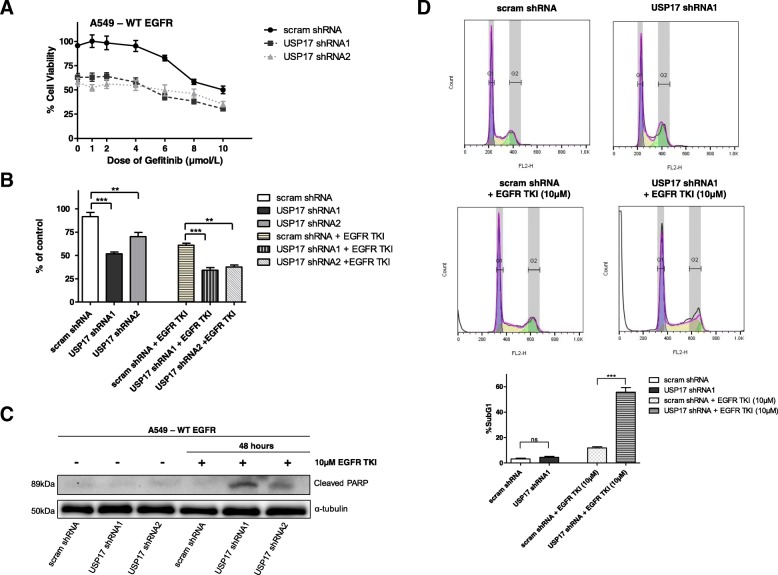
(**a**) A549 cells were transfected as indicated and 24 h after transfection cells were treated with the indicated dose of EGFR TKI (gefitinib) for 48 h. Cell viability was determined using an MTT assay and the results are plotted as % of untreated and un-transfected control. (**b**) A549 cells were transfected as indicated and 24 h after transfection, cells were treated with IC_50_ dose of EGFR TKI (10 μM) gefitinib for 48 h. Cell viability was determined using an MTT assay and the results are plotted as % of untreated and un-transfected control. (**c**) A549 cells were transfected as indicated and treated as before with the EGFR TKI (10 μM) gefitinib for 48 h. Whole cell lysates were harvested and PARP cleavage analysed by Western blotting. α-tubulin was used as a loading control. (**d**) A549 cells were transfected as indicated and treated as before with the EGFR TKI (10 μM) gefitinib for 48 h. The cells were fixed and cell cycle analysis was carried out by staining the cells with propidium iodide before assessment by flow cytometry (Upper panels). The percentage of cells in SubG1 (apoptotic cells) for each condition is plotted in a bar graph (Lower panel). ** *p* < 0.01, *** *p* < 0.001

To probe this, we transfected A549 cells as before, with either the USP17 specific shRNAs, or a non-targeting shRNA. 48 h post-transfection, the cells were treated with the indicated doses of gefitinib for a further 48 h and the viability of these cells assessed by MTT assay. USP17 depletion resulted in a significant drop in cell viability, and the combination of USP17 depletion and a range of gefitinib doses resulted in a drop in viability, compared to either gefitinib or USP17 depletion alone (Fig. 6A). This was demonstrated in particular using the combination of USP17 depletion and a 10 μM gefitinib dose which showed a significant drop in viability in the combination when compared to either alone (Fig. 6B). This indicated that USP17 depletion improves the efficacy of gefitinib in *EGFRWT* NSCLC cells. However, the previous study also indicated that blocking endocytosis in combination with gefitinib treatment could trigger a significant increase in apoptosis [24], and therefore we investigated if USP17 depletion could also do this. We transfected the A549 cells as before and immuno-blotted lysates taken after 48 h for cleaved PARP (Fig. 6C). USP17 depletion, or gefitinib treatment, didn’t have a marked impact upon the levels of PARP cleavage. However, combining USP17 depletion with gefitinib treatment resulted in a marked increase in the levels of cleaved PARP (Fig. 6C). This impact was further confirmed by cell cycle analysis using propidium iodide staining. USP17 depletion and gefitinib treatment both led to small increases in the proportion of sub G1 phase cells (apoptotic cells) (Fig. 6D). The combination of USP17 depletion and gefitinib treatment led to a significant rise in the number of sub G1 phase cells, indicating a significant rise in the proportion of cells undergoing apoptosis (Fig. 6C). These results indicated that combining USP17 depletion with gefitinib treatment markedly improved the efficacy of gefitinib in these *EGFRWT* NSCLC cells leading to an increase in cell death.

## Discussion

The data presented here demonstrates that depleting USP17 can not only block the proliferation of NSCLC cells which are *EGFRWT*, but also NSCLC cells bearing activating mutations of EGFR, as well as those with secondary gatekeeper EGFR mutations. In addition, although USP17 depletion did block CME within these cells, as demonstrated through the loss of TfR endocytosis, it did not block the endocytosis of the activated EGFR mutants (DelE746-A750 or L858R/T790M). However, USP17 depletion did alter the localization and downstream signaling of these EGFR mutants, and in particular blocked their activation of Src. As a result, USP17 depletion preferentially triggered apoptosis in NSCLC cells bearing EGFR mutations. In addition, when combined with EGFR TKI treatment, USP17 depletion could induce apoptosis in *EGFRWT* NSCLC cells, indicating USP17 inhibition can improve the efficacy and expand the susceptible patient cohort for these drugs.

USP17 is required for EGFR CME [8] and as mutant EGFRs are constitutively internalized and appear to preferentially undergo CME and recycling [22], we hypothesized that USP17 would be necessary for the endocytosis and oncogenic function of EGFR mutants. USP17 depletion did not block their endocytosis, but it did appear to alter the localization of both the DelE746-A750 and L858R/T790M mutant EGFRs to a more peri-nuclear vesicular pattern which was reminiscent of that previously observed for EGFR when high dose EGF was utilised in the absence of USP17 [8]. As a result, we propose these constitutively active EGFR mutants mimic ‘high-dose EGF’, resulting in their internalisation via non-clathrin modes of entry when USP17 is depleted.

Mutant EGFRs have been shown to display altered endocytic trafficking, undergoing ligand independent trafficking to the endocytic recycling compartment (ERC), rather than being sent to the lysosome for destruction [22, 23]. The aberrant trafficking allows them a preferential interaction with Src, which contributes to their oncogenic activity [22], and the observed loss of Src activation and proliferation, as well as the initiation of apoptosis, would indicate USP17 is vital for the aberrant trafficking of EGFR DelE746-A750 and EGFR L858R/T790M mutants. It also indicates that in the absence of USP17 these mutants do not traffic to the compartment where they interact with Src, and thus they no longer can sustain their oncogenic activity, even though the mutants are themselves still active. Indeed, this would tie in with the previous literature which has shown that Src inhibition in EGFR-dependent NSCLC cell lines results in a shutdown of the EGFR-dependent survival network and induces apoptosis [29].

There are several reported modes of non-clathrin mediated endocytosis which have been shown to be critical for attenuating EGFR signaling by trafficking EGFRs for lysosomal degradation. These include a pathway dependent on the function of the ER protein reticulon 3 (RTN3), a macropinocytic-like pathway, and fast endophilin-mediated endocytosis (FEME) [35]. It is unclear which, if any, of these pathways are utilized by the EGFR mutants in the absence of USP17, and why this doesn’t result in turnover of the receptor in the lysosome. However, it has been suggested that the L858R mutant has impaired Cbl recruitment and that this encourages hetero-dimerization with ErbB2 and hinders its trafficking to the lysosome [35]. In addition, it is unclear why we see increased EGFR, ERK1/2 and Akt activation in the absence of USP17. It has been reported that PTP1B can dephosphorylate EGFR at the recycling endosome and thus limit EGFR activation and signaling [36]. Therefore, as these mutant EGFRs undergo ligand independent trafficking to the ERC via CME [22, 23], this could explain the increase in activation, as it will block PTP1B access and thus dephosphorylation of these EGFR mutants. This would further support the theory that in the absence of USP17 these EGFR mutants fail to traffic to the ERC, something which also correlates with the loss of Src activation, as these EGFR mutants have been shown to interact with Src in the ERC [22]. In regard to the alteration in Akt levels, as well as activation, this was a consistent observation, but there is no obvious explanation and further examination of this is required to determine why USP17 impacts upon Akt stability.

Other work had previously indicated that blocking EGFR endocytosis enhances the efficacy of gefitinib towards *EGFRWT* NSCLC cells, with an endocytosis block and gefitinib combination significantly inhibiting growth both in vitro and in vivo, as well as prompting a large proportion of the cells to undergo apoptosis [24]. We hypothesized that depleting USP17 could potentially garner a similar impact upon *EGFRWT* NSCLC cells, something which was backed up by the data obtained. Indeed, depleting USP17 in combination with gefitinib had a greater impact upon *EGFRWT* NSCLC cells than either alone. Moreover, the combination of USP17 depletion and gefitinib preferentially triggered apoptosis, indicating this can enhance the efficacy of this drug in these cells.

## Conclusions

The data presented here indicates that inhibiting USP17 potentially represents a promising therapeutic strategy in NSCLC for a number of reasons. First of all, USP17 is overexpressed in NSCLC [9, 11, 13] and its expression levels are associated with poor prognosis and metastases in NSCLC [13]. Next, USP17 depletion blocks the proliferation of NSCLC cells regardless of the EGFR mutational status, indicating that USP17 inhibition could represent an opportunity to target the tumors of a large proportion of NSCLC patients. In addition, as depleting USP17 in NSCLC cells harboring EGFR activating mutations triggers their apoptosis, this indicates inhibiting USP17 may be of particular benefit in these tumors, which represent at least 10% of all NSCLC patients [17]. Also, the observation that ~ 90% of the EGFR mutants are either the exon 19 deletion, or L858R mutations [18, 19] used here, would again indicate USP17 represents an interesting therapeutic option. More interestingly, resistance to EGFR TKIs in NSCLC is mainly mediated via the acquisition of the EGFR T790M gatekeeper mutation (~ 60%) [17], and we have shown here that getting rid of USP17 can trigger apoptosis of NSCLC cells with this mutation. This would indicate targeting USP17 represents an interesting complement to EGFR TKIs, to prevent resistance, or act as an alternative once resistance is established. Finally, the observation that depleting USP17, in combination with gefitinib, triggers *EGFRWT* NSCLC cells to undergo apoptosis, indicates targeting USP17 in combination with an EGFR TKI could greatly expand the efficacy of these drugs in the NSCLC patient population.

## Abbreviations

CME: Clathrin mediated endocytosis
EGF: Epidermal growth factor
EGFR: Epidermal growth factor receptor
EGFRMT: EGFR mutant
EGFRWT: EGFR wild type
MET: Hepatocyte growth factor receptor
NSCLC: Non-small cell lung cancer
TfR: Transferrin receptor
TKI: Tyrosine kinase inhibitor
USP17: Ubiquitin specific protease 17

## References

[1] Komander D, Rape M (2012). The ubiquitin code. Annu Rev Biochem.

[2] Leznicki P, Kulathu Y (2017). Mechanisms of regulation and diversification of deubiquitylating enzyme function. J Cell Sci.

[3] Zhu Y, Pless M, Inhorn R, Mathey-Prevot B, D’Andrea a D (1996). The murine DUB-1 gene is specifically induced by the betac subunit of interleukin-3 receptor. Mol Cell Biol.

[4] Zhu Y, Lambert K, Corless C, Copeland NG, Gilbert DJ, Jenkins NA (1997). DUB-2 is a member of a novel family of cytokine-inducible deubiquitinating enzymes. J Biol Chem.

[5] Burrows JF, McGrattan MJ, Rascle A, Humbert M, Baek KH, Johnston JA (2004). DUB-3, a cytokine-inducible deubiquitinating enzyme that blocks proliferation. J Biol Chem.

[6] Burrows JF, Scott CJ, Johnston JA (2010). The DUB/USP17 deubiquitinating enzymes: a gene family within a tandemly repeated sequence, is also embedded within the copy number variable beta-defensin cluster. BMC Genomics.

[7] de la Vega M, A a K, Dunican DJ, McFarlane C, Burrows JF, Jaworski J (2011). The deubiquitinating enzyme USP17 is essential for GTPase subcellular localization and cell motility. Nat Commun.

[8] Jaworski J, de la Vega M, Fletcher SJ, McFarlane C, Greene MK, Smyth AW (2014). USP17 is required for clathrin mediated endocytosis of epidermal growth factor receptor. Oncotarget.

[9] McFarlane C, Kelvin AA, De La Vega M, Govender U, Scott CJ, Burrows JF (2010). The deubiquitinating enzyme USP17 is highly expressed in tumor biopsies, is cell cycle regulated, and is required for G1-S progression. Cancer Res.

[10] Pereg Y, Liu BY, O’Rourke KM, Sagolla M, Dey A, Komuves L (2010). Ubiquitin hydrolase Dub3 promotes oncogenic transformation by stabilizing Cdc25A. Nat Cell Biol.

[11] Zhang S, Yuan J, Zheng R (2016). Suppression of ubiquitin-specific peptidase 17 (USP17) inhibits tumorigenesis and invasion in non-small cell lung Cancer cells. Oncol Res.

[12] Zhou B, Shu B, Xi T, Su N, Liu J (2015). Dub3 expression correlates with tumor progression and poor prognosis in human epithelial ovarian cancer. Biomed Pharmacother.

[13] McFarlane C, McFarlane S, Paul I, Arthur K, Scheaff M, Kerr K (2013). The deubiquitinating enzyme USP17 is associated with non-small cell lung cancer (NSCLC) recurrence and metastasis. Oncotarget.

[14] Song Chenyang, Liu Wenge, Li Jiandong (2017). USP17 is upregulated in osteosarcoma and promotes cell proliferation, metastasis, and epithelial–mesenchymal transition through stabilizing SMAD4. Tumor Biology.

[15] Wu Y, Wang Y, Lin Y, Liu Y, Wang Y, Jia J (2017). Dub3 inhibition suppresses breast cancer invasion and metastasis by promoting Snail1 degradation. Nat Commun.

[16] Liu T, Yu J, Deng M, Yin Y, Zhang H, Luo K (2017). CDK4/6-dependent activation of DUB3 regulates cancer metastasis through SNAIL1. Nat Commun.

[17] Roskoski RJ (2014). The ErbB/HER family of protein-tyrosine kinases and cancer. Pharmacol Res.

[18] Lynch TJ, Bell DW, Sordella R, Gurubhagavatula S, Okimoto RA, Brannigan BW (2004). Activating mutations in the epidermal growth factor receptor underlying responsiveness of non-small-cell lung cancer to gefitinib. N Engl J Med.

[19] Paez JG, Janne PA, Lee JC, Tracy S, Greulich H, Gabriel S (2004). EGFR mutations in lung cancer: correlation with clinical response to gefitinib therapy. Science.

[20] Hrustanovic G, Lee BJ, Bivona TG (2013). Mechanisms of resistance to EGFR targeted therapies. Cancer Biol Ther.

[21] Lee DH (2017). Treatments for EGFR-mutant non-small cell lung cancer (NSCLC): the road to a success. paved with failures Pharmacol Ther.

[22] Chung BM, Raja SM, Clubb RJ, Tu C, George M, Band V (2009). Aberrant trafficking of NSCLC-associated EGFR mutants through the endocytic recycling pathway promotes interaction with Src. BMC Cell Biol.

[23] Nishimura Y, Yoshioka K, Bereczky B, Itoh K (2008). Evidence for efficient phosphorylation of EGFR and rapid endocytosis of phosphorylated EGFR via the early/late endocytic pathway in a gefitinib-sensitive non-small cell lung cancer cell line. Mol Cancer.

[24] Jo U, Park KH, Whang YM, Sung JS, Won NH, Park JK (2014). EGFR endocytosis is a novel therapeutic target in lung cancer with wild-type EGFR. Oncotarget.

[25] Sigismund S, Woelk T, Puri C, Maspero E, Tacchetti C, Transidico P (2005). Clathrin-independent endocytosis of ubiquitinated cargos. Proc Natl Acad Sci U S A.

[26] Van Schaeybroeck S, Kalimutho M, Dunne PD, Carson R, Allen W, Jithesh PV (2014). ADAM17-dependent c-MET-STAT3 signaling mediates resistance to MEK inhibitors in KRAS mutant colorectal cancer. Cell Rep.

[27] Mosmann T (1983). Rapid colorimetric assay for cellular growth and survival: application to proliferation and cytotoxicity assays. J Immunol Methods.

[28] Pinilla-Macua I, Grassart A, Duvvuri U, Watkins SC, Sorkin A. EGF receptor signaling, phosphorylation, ubiquitylation and endocytosis in tumors in vivo. elife. 2017;6. 10.7554/eLife.31993 PMC5741375 29268862

[29] Song L, Morris M, Bagui T, Lee FY, Jove R, Haura EB. Dasatinib ( BMS-354825 ) Selectively Induces Apoptosis in Lung Cancer Cells Dependent on Epidermal Growth Factor Receptor Signaling for Survival. 2006;:5542–5549. 10.1158/0008-5472.CAN-05-4620 16740687

[30] Alvarado-Kristensson M, Melander F, Leandersson K, Rönnstrand L, Wernstedt C, Andersson T (2004). p38-MAPK signals survival by phosphorylation of Caspase-8 and Caspase-3 in human neutrophils. J Exp Med.

[31] Yamaguchi H, Wang HG (2001). The protein kinase PKB/Akt regulates cell survival and apoptosis by inhibiting Bax conformational change. Oncogene.

[32] Cardone MH, Roy N, Stennicke HR, Salvesen GS, Franke TF, Stanbridge E (1998). Regulation of cell death protease caspase-9 by phosphorylation. Science.

[33] Zha J, Harada H, Yang E, Jockel J, Korsmeyer SJ (1996). Serine phosphorylation of death agonist BAD in response to survival factor results in binding to 14-3-3 not BCL-X(L). Cell.

[34] Reginato MJ, Mills KR, Becker EBE, Lynch DK, Bonni A, Muthuswamy SK (2005). Bim regulation of lumen formation in cultured mammary epithelial acini is targeted by oncogenes. Mol Cell Biol.

[35] Sigismund S, Avanzato D, Lanzetti L (2018). Emerging functions of the EGFR in cancer. Mol Oncologia.

[36] Baumdick M, Brüggemann Y, Schmick M, Xouri G, Sabet O, Davis L, Chin JW, Bastiaens PI (2015). EGF-dependent re-routing of vesicular recycling switches spontaneous phosphorylation suppression to EGFR signaling. elife.

